# Beyond Uncertainty: Establishing the Oda Strategy for the Treatment of Acute Aortic Dissection

**DOI:** 10.3390/jcm14155509

**Published:** 2025-08-05

**Authors:** Katsuhiko Oda, Makoto Takahashi, Ryuichi Taketomi, Rina Akanuma, Takahiko Hasegawa, Shintaro Katahira

**Affiliations:** Department of Cardiovascular Surgery, Iwate Prefectural Central Hospital, Morioka 020-0066, Japan; popeyethesailormoonjupiter@yahoo.co.jp (M.T.); ryuichi.taketomi@gmail.com (R.T.); r.akanuma224@gmail.com (R.A.); z.z.norainu@gmail.com (T.H.); shinta0911@yahoo.co.jp (S.K.)

**Keywords:** acute aortic dissection, open aortic repair, optimal medical treatment, thoracic endovascular aortic repair, false lumen expansion

## Abstract

Significant progress has been achieved in the treatment of acute aortic dissection over the past 90 years, following the first surgical intervention. This review pays tribute to the dedication of pioneers and innovators who developed advanced medical devices and therapeutic strategies to address this challenging condition. While navigating uncertainties in treatment optimization, the primary focus of the therapeutic strategies has been to save lives by increasing survival rates during the acute phase and to prevent aorta-related lethal events and late-stage thoracoabdominal aortic replacements. From a neutral standpoint, this review traces over 90 years of progress in treating acute aortic dissection. We hope that as many patients as possible will receive treatment rationally, without over- or under-treatment.

## 1. Introduction

Acute aortic dissection presents with a wide spectrum of clinical manifestations, ranging from sudden death at onset to asymptomatic cases. The diversity in symptoms and time course highlights the critical importance of tailoring appropriate treatment strategies for each patient. Although Stanford type A dissections often result in early catastrophic events involving the heart and typically require emergency surgery, Stanford type B dissections may also necessitate urgent intervention owing to complications, such as the rupture or malperfusion of visceral organs or limbs. The traditional treatment approach, which was introduced over 55 years ago, remains limited to assigning surgical interventions for type A dissections and medical interventions for type B dissections [1].

Previously, surgical options for aortic dissection relied mainly on open aortic repair (OAR), leading to poor outcomes in emergency type B dissection cases. This reinforced the reliance on the original Stanford classification (1970)-based treatment strategies. However, with the advent of thoracic endovascular aortic repair (TEVAR) in 1994 [2] and subsequent technological advancements, a third treatment option emerged, providing precise and minimally invasive management; TEVAR has become a standard modality, alongside optimal medical treatment (OMT) and OAR. Nonetheless, despite improvements in early outcomes, the management of late-phase false lumen expansion has become a primary concern. Globally, treatment strategies continue to vary, reflecting a lack of coordinated consensus.

Consistent with the principles outlined in Crossing the Quality Chasm [3], the six aims of 21st-century healthcare have gained strong advocacy. Healthcare must be

- Safe—avoiding injuries to patients resulting from the care that is intended to benefit them.
- Effective—delivering scientifically grounded services to those likely to benefit, while refraining from providing services to those unlikely to benefit, thus addressing both underuse and overuse.
- Patient-centered—providing care that aligns with individual preferences, needs, and values and ensuring all clinical decisions are guided according to patient priorities.
- Timely—reducing wait times and harmful delays for patients and their caregivers.
- Efficient—minimizing waste, particularly in the use of equipment, supplies, ideas, and energy.
- Equitable—providing care of consistent quality regardless of sex, ethnicity, geographic location, or socio-economic status.

From this perspective, the history of treatment strategies for acute aortic dissection is presented in this review.

## 2. Evolution of Aortic Dissection Treatment: Lessons from the Past

Treatment strategies for this disease over the past 90 years are summarized, emphasizing what was deemed appropriate and identifying shortcomings in practice from the perspectives of patient selection, anatomical feasibility, and long-term outcomes.

### 2.1. OMT-Only Era: Before the Advent of Surgical Options

In the era when surgical treatment for aortic dissection was unavailable, patient outcomes were dictated to chance. Data from the International Registry of Aortic Dissection (IRAD) revealed mortality rates for type A dissection at 20%, 30%, 40%, and 50% within 24 h, 48 h, 1 week, and 1 month, respectively, and patients often continued to succumb as the false lumen expanded or ruptured [4]. During this period, surgeons probably witnessed patients succumbing to the disease and assumed that mortality rates, including those for type B dissection, approached 100%. Although the progression of the disease varied, this assumption was ultimately proven incorrect [5]. The absence of computed tomography (CT) scans, challenges associated with diagnosis, and the tendency of patients with milder symptoms not to seek medical attention made it difficult for surgeons to recognize that some individuals survived. These circumstances made it logical to rely entirely on surgical intervention as the next therapeutic strategy. The world’s first surgical intervention for acute aortic dissection was reported in 1935; however, it was not until 30 years later that the first successful rescue surgery was reported [6].

### 2.2. Universal OAR Approach: The DeBakey Era

The DeBakey classification proposed that all aortic dissections should be treated surgically, regardless of whether they were in the acute or chronic phase [7]. At the time, “surgical treatment” specifically referred to OAR without the use of the frozen elephant trunk (FET) technique. This classification was proposed because the surgical techniques varied based on the type of dissection. The authors assumed that, even in type III aortic dissection, all patients would ultimately die if treated with OMT alone; therefore, it was considered logical at the time to advocate for surgical intervention for all cases. However, this assumption proved incorrect. Although their results were considered groundbreaking at the time, it later became apparent that the type III surgical outcomes were suboptimal in many centers. Moreover, only surgical outcomes were reported, while non-intervention outcomes were referenced from another study [5], casting doubt on the reliability of type III evaluations. However, their contribution as pioneers in this field is important. This era marked substantial progress in cardioplegia techniques [8,9] and cardiopulmonary bypass [10], which collectively contributed to improved cardiac surgery outcomes.

### 2.3. Stanford Classification and Its Enduring Influence

The Stanford classification of aortic dissection categorizes dissections into types A and B. A study reported for the first time that certain patients could survive without surgery [1], although it included only 35 patients. For 55 years, this disease has been treated in many facilities with straightforward strategies, such as OAR and OMT for type A and B dissections, respectively. This report, despite its limited sample size, underscores the need to reconsider the use of OAR in type B aortic dissection cases. This classification has undoubtedly brought significant benefits to the field, contributing to improved outcomes. The preference for OMT alone in type B dissections was not because future interventions were deemed unnecessary, but rather because the surgical techniques available at the time yielded poor results, making non-surgical treatment the preferred option for managing the acute phase. However, this classification may have led to the misconception that OMT alone was the best treatment strategy for type B dissections, which potentially delayed the widespread use of TEVAR intervention.

Prior to the introduction of TEVAR, type B aortic dissection was generally managed with OMT alone. Although OAR was occasionally performed in severe cases, the outcomes remained suboptimal for a considerable period.

Over time, it became apparent that the remaining false lumen expanded. To address this phenomenon, the placement of an “original elephant trunk” during initial arch replacement for type A aortic dissections was proposed in 1983, intended to facilitate future thoracic descending or thoracoabdominal replacement procedures [11].

### 2.4. Modernization of OAR: Technological Advances and New Challenges

During this period, the evolution of treatment was primarily driven by improvements in the outcomes of OAR for type A dissections. Advancements such as the development of multi-detector CT [12,13], transthoracic and transesophageal echocardiography [14], selective cerebral perfusion [15,16], sophisticated prosthetic grafts [17], various adhesives [18,19], improved anastomosis techniques [20,21], and the introduction of platelet transfusion [22], significantly contributed to better outcomes in patients presenting with type A aortic dissections.

As a result of improvements in early outcomes for patients with acute aortic dissection, it has become increasingly clear that the residual false lumen in patients post-type A aortic dissection and type B aortic dissection confers potential risks of thoracoabdominal replacement in the future. The dilemma was as follows: although ascending aortic replacement had a relatively low mortality rate, it was more prone to distant false lumen expansion. In contrast, replacement up to the aortic arch increased the mortality rate but was less likely to result in distant false lumen expansion [23,24].

### 2.5. Rise of Stent Grafts: TEVAR and FET

The first clinical application of a stent graft for an abdominal aortic aneurysm was performed in 1991 [25]. Two types of stent grafts were developed for the thoracic aortic region and were reported nearly simultaneously. These include TEVAR, which uses a catheter-based delivery system (first reported in 1994) [2], and FET, which employs a manual-based delivery system (initially referred to as “open stent graft” in the first report in 1996) [26]. FET has been actively used, particularly in Germany [27], China [28], and Japan [29], owing to its ability to be visually inserted during surgery, its simple handmade structure, and its ease of placement for OAR surgeons. However, globally, including in the aforementioned countries, OAR and TEVAR surgeons have gradually become separate teams, making it challenging to understand the advantages and disadvantages of each technique, as well as their optimal applications in different situations.

### 2.6. Clinical Acceptance of TEVAR for Complicated Type B Dissection

In the early stages, many centers were skeptical about the use of TEVAR for acute aortic dissections. Early stent grafts were primitive in design, predominantly handmade at individual facilities, with delivery systems that were thick, inflexible, and non-hydrophilic. At this stage, its use was considered a high-risk procedure. However, the use of TEVAR gained justification due to poor outcomes of OAR for complicated type B aortic dissections. Some facilities took on the challenge of employing TEVAR, and over time, its effectiveness was reported [30]. Although the initial mortality rates were relatively high, some life-saving cases were reported. Therefore, combined with revolutionary advancements in commercial device technology, TEVAR has currently evolved into the preferred first-line treatment approach for Class I indications in complicated type B acute aortic dissections [31].

### 2.7. FET: Limitations, Complications, and Ethical Concerns

There are three points to consider when evaluating stent grafts, including TEVAR and FET for aortic dissection:

1. Can stent grafts prevent false lumen expansion in cases with false lumen expansion?
2. Are unnecessary stent grafts being used in cases with no false lumen expansion?
3. Is the frequency of complications from stent grafts acceptable?

The adoption of FET during the initial OAR for acute type A dissection was driven by the hope of achieving a more extensive one-stage surgery for the thoracic aorta and improving the long-term outcomes. However, this approach lacked a solid pathological rationale from the outset. Blindly deploying a stent in a fragile, dissected aorta during the acute phase is inherently risky and has led to severe early and late complications over time. These include paraplegia [32,33], distal stent graft-induced new entry (dSINE) [34,35], and kinking [36,37] caused by spring-back force [38], malalignment, or oversizing.

Furthermore, false lumen expansion requiring intervention occurs in <30% of patients following tear-oriented surgery [39,40]. This indicates that FETs are often implanted in patients who may not have needed them, raising significant ethical concerns. In other words, more than 70% of patients who undergo tear-oriented surgery for type A aortic dissection do not experience false lumen expansion postoperatively. Therefore, even when FET is performed and deemed ‘effective’, in most cases it is merely a ‘perceived effect’, because these patients would not have developed false lumen expansion even without any intervention. However, this fact is often unrecognized.

Consequently, the routine use of FET in such cases constitutes overtreatment, unnecessarily exposing patients to potential risks. Despite these issues, certain treatment guidelines have continued to endorse these strategies, exacerbating confusion in clinical practice [31]. Further concerns arise when TEVAR is performed for complications such as dSINE following FET. Specifically, placement of an exoskeletal stent graft inside an endoskeletal FET can result in structural mismatches and dangerous gaps. The persistent endorsement of such technically flawed and ethically questionable approaches reveals a lack of critical evaluation in the development of these strategies.

### 2.8. Reassessing the INSTEAD-XL Trial: Insights into Preemptive TEVAR

Shortly after the turn of the 21st century, challenging research on preemptive TEVAR, in particular the INSTEAD-XL trial, was conducted using relatively primitive devices in patients with uncomplicated type B aortic dissection [41]. These first-generation TEVAR devices lacked adequate flexibility and compliance, which increased the risk of severe complications, including death, when applied in the fragile aortic walls during the acute or subacute phases. Furthermore, a major ethical concern was that, at the time of enrollment, it was impossible to distinguish between patients who would develop false lumen expansion and those who would not.

**Figure 1:**
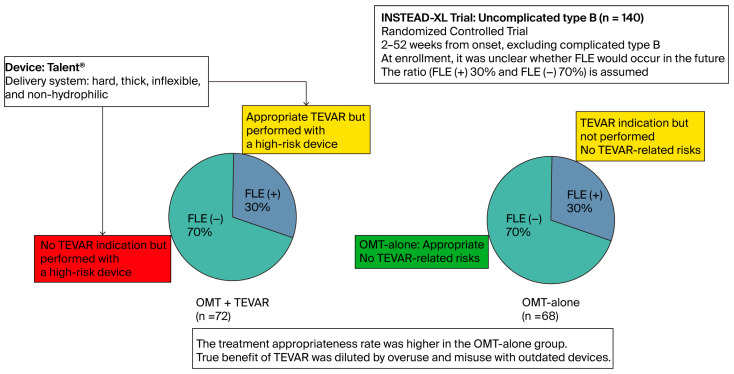
Schematic of the INSTEAD-XL trial highlighting inherent biases in the RCT design for acute aortic dissection. Red background subgroup: high-risk and unethical intervention; yellow background subgroup: potential benefits and harms; green background subgroup: appropriate treatment only. FLE, false lumen expansion; OMT, optimal medical treatment; RCT, randomized controlled trial; TEVAR, thoracic endovascular aortic repair.

Late-phase false lumen expansion occurs in only 20–50% cases of uncomplicated type B dissections [42]. To explain, we will consider the INSTEAD-XL trial, assuming that 30% patients subsequently develop false lumen expansion, which is a preemptive TEVAR indication. In this trial, 140 patients were randomized into two groups: OMT + TEVAR (72 patients) and OMT alone (68 patients). Statistically, this means that TEVAR was performed on patients who would eventually need it (30%) and those who would not (70%). Conversely, 30% of patients in the OMT-alone group did not undergo TEVAR despite requiring it, whereas 70% were appropriately managed without intervention. Consequently, appropriate treatment was administered in only 30% of patients in the OMT + TEVAR group, and 70% in the OMT-alone group (Figure 1). Therefore, the trial design was structurally biased against the OMT + TEVAR arm. Predictably, the OMT + TEVAR group showed worse outcomes during the first 2 years.

However, at 5 years, the survival benefit of OMT + TEVAR became apparent. This reversal strongly suggests that TEVAR is effective when performed on the right patients at the appropriate time. The implication is clear: if TEVAR had been performed only in the 30% of patients who genuinely required it—based on documented false lumen expansion—and using modern, flexible, and safe devices, the results would likely have been significantly more favorable. Despite its limitations, the INSTEAD-XL trial provides valuable evidence supporting the selective use of TEVAR in cases of false lumen expansion. A common misconception is that the poorer prognosis of the OMT-alone group, compared to the TEVAR group, was due to the subpar quality of OMT. Even if the quality of OMT was inadequate, its effects would have been consistent across both groups, indicating that the treatment methodology itself was not flawed.

Careful interpretation of the INSTEAD-XL trial has laid the foundation for a rational and selective treatment approach, referred to as the “Oda strategy,” which has been developed and refined in our institution since 2013. Long-term outcomes of this strategy were subsequently reported in 2022 and 2024 for type A and type B dissections, respectively [43,44]. In these papers, the authors report approximately 200 consecutive cases each of type A and B dissections and demonstrate the early and long-term outcomes of cases wherein TEVAR was performed, in addition to those wherein it was not performed, providing valuable insights into the effectiveness of these treatment selections. The Oda strategy is tailor-made and patient-centered. In line with the six aims of 21st-century healthcare [3], we reiterate that emphasis should be placed on providing necessary intervention to patients who require treatment and avoiding providing excessive intervention in patients who do not.

The basic premise of this strategy is that OMT is appropriately provided to all patients. The core principle is based on the observation that late-phase false lumen expansion occurs in only 20–30% [39,40,43] and 20–50% [42,44] of post-type A and B dissections, respectively. Appropriate patient selection is therefore crucial to improving outcomes. For both initial OAR and TEVAR in type A and B dissections, respectively, the emphasis is placed on minimizing the extent of replacement or sealing to reduce the invasiveness of procedures and complications. In our center, all cases were carefully monitored for false lumen expansion using enhanced CT imaging; TEVAR was selectively performed in patients with progressive expansion, thereby avoiding unnecessary interventions in stable patients. This rational and patient-centered approach improved clinical outcomes compared to those described in the existing literature and eliminated the need for subsequent thoracoabdominal replacement, once considered inevitable, in almost all patients (Text S1; Figures S1–S3, this document with figures serves as the Supplementary file describing the details of the Oda strategy).

Furthermore, the aforementioned trial highlights a critical challenge in conducting randomized controlled trials (RCTs) for acute aortic dissection [45]. Disease progression can significantly worsen over time, even in patients who were initially diagnosed with uncomplicated type B aortic dissection. Since randomization was performed based solely on clinical status at the time of enrollment, without the ability to predict future disease progression, patients with fundamentally different prognostic trajectories may have been assigned to the same treatment arm. Particularly, it is a serious and unignorable problem if patients who will not develop false lumen expansion in the future and for whom this cannot be predicted at enrollment, are assigned to the intervention group and consequently suffer death or major complications due to unnecessary intervention. Additionally, if such patients were assigned to the intervention group, and their false lumen did not expand, the lack of progression might falsely be attributed to the efficacy of the intervention, even though it had no actual impact. This introduces a substantial risk of overestimating the treatment effect. These issues underscore a fundamental flaw in trial design that may lead to ethically and clinically inappropriate treatment allocation, as exemplified in the current study. From this perspective, researchers planning RCTs for acute aortic dissection should fully inform patients of these potential pitfalls and obtain truly informed consent.

Therefore, from both ethical and clinical perspectives, it may be more appropriate to accumulate evidence from consecutive cases treated under a consistent individualized strategy: one that applies intervention only when clinically justified and refrains from it when not.

### 2.9. When to Intervene: Challenges in Timing TEVAR

With the exception of our studies [43,44], almost all the published reports have exclusively focused on patients who underwent TEVAR, neglecting the outcomes of those who did not. Notably, these studies fail to explain how patients without false lumen expansion were identified and excluded, thereby limiting the clinical relevance of their findings. Despite these methodological shortcomings, several reports have concluded that TEVAR yields better outcomes when performed during the subacute phase compared to the acute or chronic phases [46,47,48]. Current guidelines have also adopted this perspective [31]. For example, the VIRTUE Registry highlights that TEVAR performed in the subacute phase (15–92 days from onset) was linked to the best prognosis, while interventions performed during the acute (<14 days), or chronic (>92 days) phase were linked to poorer outcomes [46]. However, TEVAR is unnecessary in the absence of false lumen expansion. Therefore, the key clinical question is whether false lumen expansion occurs in patients without acute complications. In this context, whether a case is classified as a subacute or chronic category becomes a secondary consideration. Even if the subacute phase is deemed optimal, performing TEVAR during this window without confirmed false lumen expansion is inappropriate. Therefore, intervention is justified only when clear signs of expansion are present, even if more than 92 days have passed since onset.

### 2.10. Advances in TEVAR Technology: The GORE CTAG System

In 2017, the conventional GORE^®^ Thoracic Endoprosthesis (W.L. Gore & Associates, Inc., Flagstaff, AZ, USA) was significantly improved and reinforced in Europe, followed by the US and Japan in 2019, as the GORE^®^ TAG^®^ Conformable Thoracic Stent Graft with ACTIVE CONTROL System (GORE^®^ CTAG, W.L. Gore & Associates, Inc., Flagstaff, AZ, USA) [49,50]. Compared to conventional unsheathed-type devices, this sleeve-type device features enhanced flexibility and conformability, allowing it to adapt to the aortic curvature. The CTAG employs a staged deployment system: the primary deployment expands the device from the leading to trailing end to an intermediate diameter, followed by secondary deployment, which expands it from the trailing to leading end to its full diameter.

The primary advantage of this system is that it avoids fully occluding aorta during deployment, thereby minimizing the risk of retrograde type A aortic dissection (RTAD) due to transient hypertension in the upper body, a complication commonly associated with traditional unsheathed-type devices. Particularly, for arch TEVAR, the proximal angulation control feature is essential for achieving highly coaxial deployment, and the CTAG is currently the only device equipped with this functionality.

This technologically advanced and highly controllable device significantly outperforms the FET in terms of precision and safety. The FET is inserted blindly during OAR and is prone to being positioned in suboptimal locations, such as curved segments or near distal tears, thereby increasing the risk of complications. However, the CTAG facilitates accurate and intentional placement (Text S1; Figures S1–S3).

## 3. Future Perspectives in Aortic Dissection Management

### 3.1. OAR in the Future

OAR for acute type A aortic dissection is performed under strict time constraints, making it impractical to use prosthetic grafts that require prior preparation, such as tissue-engineered vascular grafts [51]. It was originally developed for small-caliber vessels and lacks the durability required for high-pressure regions such as the thoracic aorta. Among the three treatment options, namely OAR, TEVAR, and OMT, OAR remains the most technically mature. Further innovation is expected.

### 3.2. Next-Generation TEVAR: Opportunities and Limitations

Currently, the GORE CTAG remains the most suitable TEVAR device for this condition, although further refinements, such as reducing the device profile to facilitate accessibility, could be beneficial. Branched devices, such as Thoracic Branch Endoprosthesis (TBE^®^, W.L. Gore & Associates, Inc., Flagstaff, AZ, USA), are emerging as alternatives that may reduce the need for surgical debranching [52]. However, device enhancements often come with trade-offs. The TBE^®^ lacks the proximal angulation control mechanism found in the CTAG. Additionally, all unsheathed-type devices currently face the critical drawback of completely occluding the aortic lumen upon deployment, which remains a pressing issue that requires immediate attention.

TEVAR for type A aortic dissections poses significant technical and physiological challenges. A particularly critical issue is the considerable variation in the diameter of the ascending aorta between the systolic and diastolic phases. Therefore, any future TEVAR device designed for this region must accommodate these dynamic changes in vessel size. Although global efforts to develop TEVAR for type A aortic dissections are ongoing, clinical application remains a distant prospect [53,54].

### 3.3. Emerging Therapies in OMT: Pharmacologic and Cellular Innovations

The foundation of drug therapy in OMT involves antihypertensive medication; however, new agents, such as zilebesiran, which act via novel mechanisms, may emerge in the future [55]. Given the fragility of the aortic wall, particularly the intima, which is considered a key factor in primary entry formation, efforts are underway to develop pharmacological therapies aimed at reinforcing the structural integrity of the aortic wall. For example, the transforming growth factor-beta signaling pathway and matrix metalloproteinase activity have been identified as promising therapeutic targets [56,57]. To prevent rupture, new approaches that specifically act on the adventitia to enhance tissue strength, such as the use of multilineage-differentiating stress-enduring cells [58] and nanocarrier-based delivery systems [59], are being explored. The efficacy of these treatments has been reported in experimental animal models.

## 4. Conclusions

Ultimately, the future of aortic dissection treatment depends on scientifically sound and ethically justified strategies. In line with the six aims of 21st-century healthcare, we reiterate that emphasis should be on providing necessary intervention to patients who need it, while avoiding providing excessive intervention to patients who do not need it.

## Supplementary Materials

The following supporting information can be downloaded at https://www.mdpi.com/article/10.3390/jcm14155509/s1, Text S1: Comprehensive strategy for maximizing treatment effectiveness and minimizing risk in acute aortic dissection; Figure S1. Treatment algorithm of the strategy for maximizing effectiveness and minimizing risk; Figure S2. Assessment of false lumen expansion (FLE) and postoperative course after TEVAR; Figure S3. Aortic dissection progression and TEVAR timing based on the aortic hiatus [60,61,62,63,64,65,66,67,68,69,70].

## Abbreviations

The following abbreviations are used in this manuscript:

CT	Computed tomography
CTAG	Conformable thoracic aortic graft
dSINE	Distal stent graft-induced new entry
FET	Frozen elephant trunk
FLE	False lumen expansion
IRAD	International Registry of Aortic Dissection
OAR	Open aortic repair
OMT	Optimal medical treatment
RCT	Randomized controlled trial
RTAD	Retrograde type A aortic dissection
TBE	Thoracic branch endoprosthesis
TEVAR	Thoracic endovascular aortic repair
