# Supplementary material for: Beyond Uncertainty: Establishing the Oda Strategy for the Treatment of Acute Aortic Dissection
Source: J Clin Med. 2025 Aug 5;14(15):5509. doi: 10.3390/jcm14155509 (PMC12347240; doi:10.3390/jcm14155509)
Supplement: Supplementary file 1 [file jcm-14-05509-s001.zip › jcm-3680447-supplementary.pdf]

Journal of
Clinical Medicine

Text S1

The Oda strategy: A Comprehensive Strategy for Maximizing Treatment Effectiveness and Minimizing Risk in Acute Aortic Dissection

Katsuhiko Oda *, Makoto Takahashi, Ryuichi Taketomi, Rina Akanuma, Takahiko Hasegawa and Shintaro Katahira

Department of Cardiovascular Surgery, Iwate Prefectural Central Hospital, Morioka, Japan
* Correspondence: oda2015@gmail.com; Tel.: +81-19-653-1151

Keywords: acute aortic dissection; open aortic repair; optimal medical treatment; thoracic endovascular aortic repair; false lumen expansion

Academic Editor(s): Name

Received: date
Revised: date
Accepted: date
Published: date

Citation: Oda, K. Text S1—Comprehensive Strategy for Maximizing Treatment Effectiveness and Minimizing Risk in Acute Aortic Dissection. *J. Clin. Med.* **2025**, *14*, x. https://doi.org/10.3390/xxxxx

Copyright: © 2025 by the author. Submitted for possible open access publication under the terms and conditions of the Creative Commons Attribution (CC BY) license (https://creativecommons.org/licenses/by/4.0/).

1. The Core Principle and Treatment Algorithm

As stated in this review, acute aortic dissection is a condition that presents diverse pathologies and symptoms; appropriate treatment selection for each patient is therefore vital. Careful interpretation of the results of the INSTEAD-XL trial has laid the foundation for a rational and selective treatment approach that has been developed and refined since 2013, as reported in previous studies [43,44]. In these papers, the authors report approximately 200 consecutive cases each of type A and B dissections and demonstrate the early and long-term outcomes of cases wherein thoracic endovascular aortic repair (TEVAR) was performed, in addition to those wherein it was not performed, providing valuable insights into the effectiveness of these treatment selections. This strategy is tailor-made and patient-centered. In line with the six aims of 21st-century healthcare [3], we reiterate that emphasis should be placed on providing necessary intervention to patients who require treatment and avoiding providing excessive intervention in patients who do not.

The basic premise of this strategy is that optimal medical treatment (OMT) is provided appropriately to all patients. The core principle is based on the observation that late-phase false lumen expansion occurs in only 20–30% [39,40,43] and 20–50% [42,44] of post-type A and B dissections, respectively. Appropriate patient selection is therefore crucial for improving outcomes. For both initial open aortic repair (OAR) and TEVAR in type A and B dissections, respectively, emphasis is placed on minimizing the extent of replacement or sealing to reduce the invasiveness of procedures and complications. In our center, all cases were carefully monitored for false lumen expansion using enhanced computed tomography (CT) imaging; TEVAR was selectively performed in patients with progressive expansion, thereby avoiding unnecessary interventions in stable patients. This rational and patient-centered approach improved clinical outcomes compared to those described in the existing literature and eliminated the need for subsequent thoraco-abdominal replacement, once considered inevitable, in almost all patients (Figure S1). Our surveillance schedule is as follows:

- Enhanced CT is performed at the onset, and then at 1, 2, and 3 weeks, followed by 1 month after onset.
- After that, routine CT scans are scheduled at 3 months, 6 months, and 1 year.

- In total, 8 CT scans are typically performed during the first year.
- Additional CTs are only performed as needed, for example, in cases with suspected false lumen expansion or new symptoms.
- In addition to the 8 enhanced CT scans performed during the first year, we continue to routine surveillance imaging thereafter. Specifically, enhanced CT is performed every 6 months for the following two years (i.e., up to 3 years after onset), and then annually from the fourth year onward.

This schedule allows for early detection of progressive false lumen expansion and timely intervention to prevent thoracoabdominal aortic replacement.

2. Definition of False Lumen Expansion and How to Measure it

Although several risk factors associated with an increased likelihood of false lumen expansion have been reported [31], these factors primarily include quantifiable measurements such as tear size, number, and single-point diameter measurements. However, these factors are considered as reference information only, as the fragility of the adventitia and lifestyle management, which are the most important factors influencing false lumen expansion, cannot be quantified. Ultimately, there is no alternative to directly measuring and confirming false lumen expansion over time through enhanced CT imaging.

TEVAR is performed exclusively in patients with confirmed false lumen expansion, defined as >5 mm over 6 months [43,44]. Since intimal tears can occur at any location unexpectedly, expansion should be assessed across the entire thoracic aorta and in all directions (axial, sagittal, or coronal). Specifically, the appropriate location and view for evaluating false lumen expansion differ between patients (Figure S2). False lumen expansion is often only visible in the long diameter direction, and thus, measuring the maximum short diameter is meaningless, because this condition is completely different from true aneurysms. Furthermore, even if no difference in the outer diameter is noted, it is important to exercise caution in cases in which the proportion of the false lumen increases and that of the true lumen becomes narrower.

Next, we accurately identify the location of the tear that contributes to false lumen expansion. During the acute phase, it is difficult to precisely identify the tear location because the intimal flap flutters; however, this location gradually becomes clearer from the subacute phase onwards. Once the tear contributing to false lumen expansion has been identified, TEVAR is performed so that the tear can be adequately closed, and both ends are positioned appropriately. The need for debranching of the left common carotid and the left subclavian arteries and for coiling of the left subclavian artery is also considered.

Furthermore, the rate of false lumen expansion (>5mm/6 months) is important. This factor does not mean that we should simply wait 6 months before evaluation; if there is an expansion of >2.5 mm in 3 months or >1 mm in 1 month, it should be noted that there is a likelihood of false lumen expansion. This information should be shared with the patient, and OMT should be performed thoroughly. Once the condition for false lumen expansion is met, TEVAR should be performed promptly; however, it is also important to share information on the likelihood of false lumen expansion with patients and to encourage them to take measures to avoid it being necessary for TEVAR to be performed.

It is also important to recognize that TEVAR is not necessary in patients without false lumen expansion, which occurs in 70–80% [39,40,43] of uncomplicated post-type A and 50–80% [42,44] of uncomplicated type B patients.

3. The Role of the Aortic Hiatus and the Timing of TEVAR

One puzzling aspect of the INSTEAD-XL trial was the fact that TEVAR was only performed on the thoracic descending aorta, yet there was little need for subsequent

interventions on the abdominal aorta. The authors of certain reports [43,44] were among the earliest to highlight the anatomic role of the aortic hiatus, through which the aorta passes the diaphragm, as a key factor affecting the long-term prognosis of acute aortic dissection (Figure S3). This important observation gives us confidence in the validity of this strategy. If the false lumen pressure above the aortic hiatus does not decrease owing to patent tears, the false lumen of the thoracic descending aorta continues to expand. If the tear is properly closed in a timely manner and the false lumen pressure decreases, the true lumen pressure may increase accordingly and the lumen size will naturally normalize.

We have previously reported differences in the feasibility of false lumen expansion between the thoracic descending aorta and abdominal aorta. When tears were present only in the thoracic descending aorta, TEVAR was performed in 93% of cases; however, when tears were present only in the abdominal aorta, TEVAR was not necessary. The authors of one previous study speculated that the mechanism responsible for this phenomenon may be the difference in the surrounding structures. Almost no tissue is present around the thoracic descending aorta; in contrast, the abdominal aorta, particularly its upper section, is intertwined with lymph nodes and plexus, and it possesses large branches, such as the celiac trunk and superior mesenteric artery. The study authors also speculated that blood flow from the tear flows into the branches originating from the false lumen, making it difficult for the false lumen pressure to increase. It is expected that the authors of future studies will examine this hypothesis in further detail.

4. Creating a System for Timely Therapeutic Intervention

Type A and B aortic dissections are managed from onset by cardiovascular surgeons who oversee the case from diagnosis and determine the optimal timing for intervention. To enable effective implementation of this approach, proficiency in both OAR and TEVAR is essential. Surgeons skilled in both procedures can make informed decisions and tailor treatment plans to each patient's unique pathology. If OAR and TEVAR are performed by different teams, close coordination between said teams becomes necessary. Future generations of thoracic aortic specialists must be trained in both skill sets to uphold the highest standards of care.

In outpatient and emergency settings, the ability to examine patients with aortic dissection is vital. The cardiovascular surgery team should implement a rapid decision-making process from diagnosis to treatment. In our department, the division of roles within the team is clearly defined [60].

In addition to the above measure, a regional network has been established to ensure timely referrals. In Iwate Prefecture, the institution has established formal communication with more than 1,600 primary care and cardiology centers, emphasizing the importance of promptly transferring both type A and type B cases to the appropriate surgical team. This systematic outreach has contributed to optimized patient outcomes and reduced delays in care.

5. Building an In-Facility Database for Acute Aortic Dissection and Data Analysis

Every institution generally has and maintains a database of patients with type A aortic dissection who have undergone surgery. However, patients with type A or B aortic dissection who have not undergone surgery are often not registered; however, it is imperative that such patients be registered. It is necessary to register all patients with this condition, including those who have not undergone OAR or TEVAR, and to evaluate the appropriateness of treatment choices in both patients who have undergone surgery and

those who have not. One of the most important aspects of our previous study [44] is the fact that it demonstrated that the long-term prognosis was equally good for both patients who had undergone TEVAR and those for whom it was not necessary.

We used Claris FileMaker Pro, latest version (Claris international Inc., Cupertino, CA, USA), to build our database; after receiving approval from the Institutional Review Board, all statistical analyses are performed using JMP, latest version (SAS Institute, Inc., Cary, NC, USA), with consultation with statistical experts.

6. Importance of Anti-Hypertension and Lifestyle Management

OMT includes both pharmacological treatments, primarily antihypertensives, and lifestyle modifications. Ongoing patient adherence to blood pressure control and healthy habits is vital in preventing false lumen expansion. Patients prone to false lumen expansion, even during the subacute phase, often experience cessation of this progression after adopting improved lifestyle practices, such as avoiding heavy lifting (>10 kg), reducing their workload, and taking measures to prevent constipation. These anti-hypertensive and lifestyle improvements are especially important if there is a tear in the lower part of the thoracic descending aorta or shaggy aorta [61] and there is a risk of paraplegia with TEVAR intervention.

7. Respect for the Patient's Right to Self-Determination

In accordance with the six aims of 21st-century healthcare [3], when patients make treatment decisions, they are given a full explanation of the disease/condition and treatment methods, including OAR, TEVAR, OMT, and their combination, and are presented with different treatment options, including information on the consequences of not treating the disease/condition and the necessity and importance of each treatment modality for each individual patient. The families of patients are also entitled to a full explanation of the above.

8. Technical Aspects of Initial OAR

In our patients, we selected the tear-oriented surgery to minimize the mortality and morbidity rates. By minimizing the risks of the initial OAR and performing subsequent TEVAR only in patients with residual false lumen expansion, we are able to rationally avoid overtreatment in the initial OAR (Figure S1). As shown in a previous report [43], the mortality rate of initial OAR for acute type A aortic dissection was excellent (8.3%, compared to 15–18% in EACTS/STS guidelines) [31]. Another notable feature is the fact that various considerations are made during the initial OAR to enable TEVAR, which may subsequently be performed.

8.1. Preoperative Planning and Preparation

Important checkpoints in CT imaging include the location of the primary entry, identifying arteries capable of reliably supplying blood to the true lumen (primarily the right axillary and femoral arteries), and locating the arch branches from a technical perspective. Clinically, it is essential to confirm the presence or absence of cerebral infarction and cardiac tamponade. Patients in a coma are generally considered unsuitable for surgery. In addition, transthoracic and transesophageal echocardiography play a vital role in assessing cardiac contractility, valvular function, and cardiac tamponade. If a coronary artery occlusion is suspected, particularly with left coronary artery involvement, cardiologists should be consulted to promptly perform percutaneous coronary intervention to restore coronary blood flow.

If necessary, coronary artery bypass grafting is initiated. Considering future intervention in the distal aorta, the left internal thoracic artery should not be used to avoid the potential risk of paraplegia, and free grafts such as the great saphenous vein should be used instead. In the initial OAR for type A dissection, we routinely use the frog leg position because there is a possibility of coronary artery bypass grafting, including unforeseen circumstances.

8.2. Establishment of Cardiopulmonary Bypass (CPB) and Selective Cerebral Perfusion (SCP)

If cardiac tamponade is present, it is necessary to decide whether to relieve it first or to secure the sites for arterial return. Appropriately sized prosthetic grafts (J-Graft®, Japan lifeline Co., Ltd., Tokyo, Japan) are anastomosed to arteries that reliably supply blood to the true lumen, ensuring at least one for both the upper and lower halves of the body, and then cannulated. We avoid direct ascending aortic cannulation due to the potential risk of malperfusion into the false lumen through the primary entry tear. Even when guided by epi-aortic or transesophageal echocardiography, we believe this approach may not ensure adequate patient safety. Therefore, we prefer peripheral arterial perfusion using intact axillary or femoral arteries. To prevent retrograde dissection, the artery is not cannulated directly. If lower limb ischemia is suspected, blood infusion should be directed to secure the true lumen while also delivering blood to the ischemic side to address lower limb ischemia when CPB is established. Venous drainage is usually established using a two-stage cannula via the right atrial appendage. The cardiopulmonary bypass system utilized at our center is Stockert S5® (LivaNova Deutschland GmbH, Munich, Germany). Heparinization during CPB includes a 0.35 mL/kg bolus, and the activated clotting time is maintained at >480 s.

In addition, because the common femoral arteries can potentially be used as an access site for TEVAR, we attempt to preserve and not expose the area immediately below the inguinal ligament.

For the right SCP, the SP-GRIPFLOW™ (12Fr, Fuji Systems Corporation, Tokyo, Japan) cannula is inserted via a prosthetic graft anastomosed to the right axillary artery, and the brachiocephalic artery is clamped before the open distal anastomosis to establish the SCP. For the left SCP, 12 Fr SP-GRIPFLOW™ cannulas are inserted into the left common carotid artery and left subclavian arteries during the open distal anastomosis. Bilateral SCPs were managed in a previous study using independent roller pumps to maintain a constant flow rate (right: 5 mL/kg/min, left: 6.5 mL/kg/min), with the aim of preventing watershed cerebral infarction [16]. Cerebral blood flow can be monitored using regional oxygen saturation with NIRO-200NX (Hamamatsu Photonics K.K., Hamamatsu, Japan) and cannula tip pressure.

8.3. Considerations for Clamping the Ascending Aorta

If the false lumen in the ascending aorta is thrombosed, the ascending aorta is not clamped, and the procedure begins with open distal anastomosis. Conversely, if the false lumen is patent, the ascending aorta is clamped, and the procedure begins with proximal anastomosis. If the false lumen is thrombosed and clamped, there is a risk of thrombus dislodgement, potentially migrating through the tear into the left coronary artery. When the proximal anastomosis is prioritized, the stump is created, followed by prosthetic graft anastomosis, facilitating subsequent myocardial preservation through the graft (J-Graft®) and minimizing the need for direct coronary artery cannulation.

8.4. Proximal Aortic Anastomosis

It is important to ensure that there is acceptable aortic valve function and no residual false lumen at the aortic root following the initial OAR procedure. Injection of adhesive

between the intima and adventitia is not performed or is kept to a minimum. The original anastomotic technique that we adopted involves cuffed anastomosis [20]. Preventing tear formation due to needle holes during stump preparation is of paramount importance. To address this issue, a specialized felt (Feltina Pro®, Kono Seisakusho Co., Ltd, Chiba, Japan) has been developed to facilitate a safe stump preparation method. A 1 cm wide aortic stump is created using the aortic wall with the sino-tubular junction at the lower end using a ring of Feltina Pro, and the left and right coronary artery ostia are confirmed to be open. It is important that the intimal and adventitial edges extend at least 1 mm beyond the upper edge of the inner and outer reinforcing felt strips. This process prevents inadvertent exclusion of the intima or adventitia when suturing them to the prosthetic graft. In addition, it is essential to secure the felt with sutures at six points: each commissure and the midpoint between commissures. The results of this approach will be reported in the future.

This proximal stump formation method can aid in the control of aortic valve regurgitation caused by aortic dissection; however, because significant aortic valve regurgitation present before disease onset often remains, the replacement of the valve with a prosthetic version is performed immediately. If necessary, an aortic root replacement is also performed.

8.5. Distal Aortic Anastomosis

The distal anastomosis is performed under hypothermic conditions, with ascending and arch replacement performed at 22 °C and ascending replacement performed at 25 °C, measured based on tympanic membrane temperature, combined with low distal perfusion (300–500 mL/min). The extent of replacement is determined by the location of the primary entry tear (tear-oriented surgery). If it is in the ascending aorta, an ascending replacement is performed. When preoperative CT imaging estimates tears that are not visible during surgery, an ascending and partial arch replacement is performed with reconstruction of the brachiocephalic artery to prepare for future TEVAR. If a visible tear is identified in the arch, an ascending and total arch replacement is performed.

In addition, we never use FET to prevent overtreatment because it is impossible to identify patients who will develop false lumen expansion during the initial OAR. For arch branch reconstructions, a distance of 4 cm is maintained from the site of the most peripheral side branch to the distal anastomosis site to ensure a landing zone for TEVAR.

As mentioned in the section on proximal aortic anastomosis, it is also important that the intimal and adventitial edges extend at least 1 mm beyond the upper edge of the inner and outer reinforcing felt strips. These measures prevent inadvertent exclusion of the intima or adventitia when suturing them to the prosthetic graft. Of late, even when performing total arch replacement, the original elephant trunk is no longer used because it may interfere with subsequent TEVAR.

After the distal aortic anastomosis is completed, the arterial return is switched from the common femoral artery to the most distal of the four branches of the prosthetic graft (J-Graft®). The volume of arterial return should be 500 mL/min during air evacuation and connection of the arterial return cannula with the branch. If it is higher than this value, there is a risk of rupture if false lumen perfusion occurs via the distal tears; care must therefore be taken. This graft side branch is finally triple-ligated at its base to prevent the formation of any pouch-like structures. This side branch is not included in the measurement to ensure a distance of 4 cm from the distal aortic anastomosis. The arch branches are reconstructed with the three more proximally positioned graft branches.

8.6. Reconstruction of Arch Branches and the Brachiocephalic Flap Technique

In most cases, reconstruction of the arch branches is performed after aortic reconstruction is completed and the heart is reperfused; however, reconstruction of the deep left subclavian artery is performed simultaneously with open distal aortic anastomosis. A 5 mm wide Feltina® (Kono Seisakusho Co., Ltd., Chiba, Japan), which was developed based on a concept from our department, is used to reinforce the anastomosis in outer fashion.

In partial arch replacement, reconstructing only the brachiocephalic artery, the brachiocephalic artery and the left common carotid artery are often in close proximity, necessitating ingenuity during reconstruction. In such cases, the subclavian flap technique used in congenital surgery for coarctation of the aorta is adapted [62]. Reconstruction is performed using the brachiocephalic flap technique and a two-branched prosthetic graft (J-Graft®). We plan to report the outcomes of this approach in the future.

8.7. Weaning from the CPB and Hemostasis Technique

Once the tympanic temperature has returned to 35 °C, weaning from the CPB begins. Air evacuation and venting from the left ventricle are important during weaning from the CPB. When weaning from the CPB begins, the head-down position should be assumed, and when the amount of arterial return is reduced by half, the bed should be flattened. If the bed is flattened after removal of the CPB, the remaining air may enter the right coronary artery and induce a fatal arrhythmia. Once there is no air inside the heart (confirmed through transesophageal echocardiography) and sufficient cardiac function has recovered, the patient is weaned from the CPB.

If the patient presents with good cardiac function, no particular difficulties should arise. If their cardiac function is impaired, we consider Impella® (ABIOMED®, Danvers, MA, USA) support. The standardized hemostasis technique used in the department was established in 2011, and the outcomes are currently being submitted for publication in a medical safety journal. The re-exploration rate for bleeding has been <1% for the past decade.

9. Technical Aspects of TEVAR for Post Type A and B Dissection

We aimed to minimize the extent of TEVAR sealing to reduce the invasiveness of the procedure and complications. For patients with false lumen expansion, TEVAR should be performed in a timely manner to avoid undertreatment, which could result in missing the opportunity to treat such patients. Based on the results of a previous report [44], the mortality rate during hospitalization at the onset of acute type B was found to be excellent (3.1%, compared to 14.1% in EACTS/STS guidelines) [31]. At the institution in question, the mortality rate of TEVAR was as follows: 0% for post-type A, 3.7% for acute complicated type B, and 0% for uncomplicated type B during subacute and chronic phases [43,44].

Interestingly, the findings of the most recent International Registry of Aortic Dissection (IRAD) report demonstrated that the implementation rate of TEVAR for type B patients was 37.2% over a 5-year period (September 2017–August 2022) [63]. In this study [44], in which the indications for TEVAR are clearly defined based on complicated type B cases and progressive false lumen expansion (>5 mm/6 months), the corresponding rate was 40%. Although certain variations in TEVAR indications across IRAD-participating institutions are to be expected, this finding strongly suggests that the proportion of type B patients genuinely requiring TEVAR may align at approximately 40%.

9.1. Preoperative Planning

Identifying patients with complicated type B dissection is relatively straightforward, as most present with severe symptoms, such as hypotension, severe back pain, and

abdominal or lower limb pain. However, accurately identifying patients with significant false lumen expansion among uncomplicated post-type A and type B cases remains challenging. The need for TEVAR varies depending on the morphological characteristics of type B dissection, and we plan to report the findings in this regard in the future.

It is essential to identify the site and direction where false lumen expansion is most evident, as this can vary between patients (Figure S2). In addition to appropriate size (identical to the longitudinal diameter of the true lumen; 100% oversizing rate to the native aorta) and length, an important consideration when planning TEVAR is to place the proximal end in a position that does not receive blood flow from the outside of the graft (one of the reasons for retrograde type A aortic dissection [RTAD]); furthermore, the distal end should not be placed in a curved region that might cause distal stent graft-induced new entry (dSINE). TEVAR planning is conducted using OsiriX® MD (Pixmeo SARL, Geneva, Switzerland).

9.2. Details of TEVAR Timing

In the case of complicated type B aortic dissection, TEVAR should be performed promptly; however, performing TEVAR during the acute phase should be avoided where possible for uncomplicated post-type A and B aortic dissection [64]. After the initial OAR for DeBakey III retrograde, even if a primary entry tear remains, TEVAR is not performed during the acute phase and is instead performed during the subacute phase or later if false lumen expansion is confirmed. In cases of uncomplicated post-type A, we believe that TEVAR should be avoided in the acute phase to promote recovery from minor organ damage caused by open distal anastomosis.

Conversely, we should avoid performing TEVAR at too late a stage (>3 years from onset), because at this time, the aortic hiatus has often already dilated, and thoracoabdominal replacement is often inevitable. As a general rule, we aim to complete the identification of patients with or without false lumen expansion within 1 year from the onset of acute aortic dissection. Furthermore, an ulcer-like projection, a feature of a case initially described in Japan, represents a localized re-expansion of the thrombosed false lumen. In this lesion, even 3 years after onset, the aortic hiatus does not enlarge and expands the false lumen locally; thus, TEVAR is performed if false lumen expansion is confirmed.

9.3. Debranching

For debranching, one option from the following three bypass options is chosen: the right axillary artery–left axillary artery bypass, the left common carotid artery–left subclavian artery bypass, and the right axillary artery–left common carotid artery–right axillary artery bypass. The first two options are for Zone 2 landing, and the third option is for Zone 1 landing.

In cases of complicated type B dissection, due to emergency, the tear may be closed using TEVAR before debranching; however, in most cases, debranching using 7 or 8 mm GORE® PROPATEN® (W.L. Gore & Associates, Inc., Flagstaff, AZ, USA) and CV-6 (needle-to-thread ratio 1:1, W.L. Gore & Associates, Inc., Flagstaff, AZ, USA) is performed first if necessary under heparinization (0.15 mL/kg bolus; maintaining an activated clotting time at >250 s), followed by TEVAR. Although maintaining blood flow in the left common carotid artery is of clear importance, ensuring sufficient blood flow in the left subclavian artery is equally critical. Such measures are necessary to prevent paraplegia by sustaining collateral circulation from the left internal thoracic artery, in addition to preventing thrombus formation by maintaining antegrade blood flow to the left vertebral artery [65–67]. To achieve this aim, the graft anastomosis to the axillary artery must be performed vertically. In addition, after debranching prior to TEVAR, heparin is administered to

prevent occlusion of the graft due to flow competition towards the antegrade flow of debranched vessels.

When anastomosis is performed between the left common carotid artery and the prosthetic graft, it is necessary to clamp the former. While monitoring regional oxygen saturation, a clamp test for the left common carotid artery is performed. If there is no sudden drop in left regional oxygen saturation, the anastomosis is continued. However, if left regional oxygen saturation immediately drops, the clamp is released, and blood flow is resumed. Next, a 12 Fr SP-GRIPFLOW™ cannula is connected to the branch for the left axillary artery that has already been anastomosed to the right axillary artery, and the anastomosis is performed under distal perfusion of the left common carotid artery. The frequency of the need for distal perfusion into the left common carotid artery is 7% in our case series.

In post type A, a landing zone of approximately 4 cm is created in the prosthetic graft immediately preceding the distal anastomosis; as a result, the frequency of debranching during TEVAR is low.

Furthermore, to maintain focus during procedures, operators perform this step without radiation-protective equipment. To enable precision surgery, tools such as the Castroviejo needle holder and 2.8 mm puncher for coronary anastomosis are utilized. Following the completion of debranching, the TEVAR operator and assistants wear radiation-protective equipment; in addition, the operator wears lower leg shielding [68].

9.4. Access Site

The femoral and iliac arteries in patients with this condition are fragile, making it essential to strictly avoid inserting devices that are too large. If the external iliac artery is small, the common iliac artery is exposed via a retroperitoneal approach. In such a case, SurgiSleeve™ (size S or M, Medtronic plc, Galway, Ireland) is used as a wound retractor that possesses radiolucency.

9.5. Guide Wires and Digital Subtraction Angiography System

Wire handling is performed with great care. After switching from Angled Radifocus® Guide Wire M (TERUMO CORPORATION, Tokyo, Japan) to Lunderquist® Extra-Stiff Wire Guide (Cook Medical Inc., Bloomington, IN, USA), forceful methods such as pushing the wire into the greater curvature of the arch, which may cause unexpected vascular or left ventricular injury and RTAD, are strictly avoided. A digital subtraction angiography system (DSA, ARTIS icono biplane angiography system, SIEMENS, Erlangen, Germany) is employed in hybrid operating rooms.

Furthermore, to prevent deviation or dislodgement during delivery, the use of relatively soft wires, which pose safety risks, is avoided. Instead, the Lunderquist® Extra-Stiff Wire Guide is routinely used for TEVAR in aortic dissection cases to ensure patient safety.

9.6. Searching for the True Lumen Using Intravascular Ultrasound (IVUS)

The optimal method for exploring the true lumen involves using the Angled Radifocus® GuideWire M in combination with IVUS (Vision PV .035, IntraSight Mobile, Royal Philips, Amsterdam, The Netherlands) [69]. Even a short portion of the guidewire passing through the false lumen can lead to serious injury; thus, all members of the surgical field must carefully examine IVUS images to ensure that the guidewire is in the true lumen along its entire length in the patient's body.

9.7. Delivery and Deployment

GORE® CTAG is a revolutionary device that reduces blood flow resistance during deployment [49]. Typically, when the device is folded, it offers little resistance to blood

flow; however, as it begins to unfold, the device usually creates greater resistance to blood flow and is washed away. We have a full comprehension of fluid mechanics and take due care in deploying the device in the correct position. In the arch, blood flow runs horizontally, and the delivery system possesses a vertical portion; therefore, unless the resistance of the device is reduced during deployment or the delivery system has high rigidity, the device tends to flow to the periphery.

As a general rule, the first piece inserted should close the target tear [44]. If the target tear remains open and the first piece is placed distally, the false lumen at that site will be temporarily compressed, increasing blood flow resistance. This factor raises the risk of the dissection progressing proximally, known to lead to RTAD. In addition, if the true lumen is narrowed to the same size as the device during delivery, there is a risk of the true lumen being closed, causing a sudden increase in upper body blood pressure, which can lead to RTAD. In such cases, prompt retraction of the device is performed, and delivery is carried out slowly at a rate that allows the anesthesiologist to manage any increased response in blood pressure effectively. Devices such as the GORE® CTAG are advantageous as they enable adjustment to the proximal end's angle during the initial deployment; subsequently adjusting the angle could increase the risk of RTAD and should be avoided. Furthermore, it is critical to ensure that the device is not exposed to external blood flow after placement (avoiding the so-called bird beak), as such a situation could lead to new tears on the opposite side of the area exposed to external blood flow. In addition, transient balloon occlusion of the left subclavian artery during the delivery and deployment of TEVAR is performed to prevent embolism in patients with a shaggy aorta [70].

9.8. Examining the Access Route After the Procedure

After completing the primary procedures, angiography is performed to assess for damage to the RTAD at the ascending aorta and access route. It also aids in the examination of the state of backward flow in the false lumen through the aortic hiatus and the perfusion status of the abdominal branches and lower extremities.

Due to the potential risk of injury to the iliac arteries, the sheath is kept within the wire rather than being fully removed during its withdrawal from the common femoral artery. In the event of a sudden drop in blood pressure, the sheath is promptly reinserted into the abdominal aorta. This action seals the damaged area of the iliac arteries, providing time to prepare the necessary equipment for repair.

10. Predictable and Unpredictable Additional Interventions

As indicated in one report [43], additional arch replacement was performed following initial OAR for type A aortic dissection in patients who presented with tears in the arch branches, such as the brachiocephalic and left subclavian arteries. More recently, CT imaging has enabled the identification of tears in the arch branches, leading to partial or total arch replacement being performed to prevent blood flow into the false lumen of the aorta through the branches.

For cases involving multiple tears in the thoracic descending aorta [44], the aim of the initial TEVAR is to close the largest tear, resulting in a minimal sealing area to prevent paraplegia. Additional TEVAR is undertaken only if false lumen expansion persists due to another tear, which is considered a predictable intervention. Conversely, after TEVAR is performed in the acute phase, the extremely narrow true lumen may be drastically normalized, leading to stent graft migration. This factor necessitates additional OAR or TEVAR several months later due to complications such as RTAD and dSINE. Such interventions are unpredictable. Following TEVAR, particularly for complicated type B dissections during the acute phase, close follow-up and timely intervention remain essential [64].

Even among patients who have suffered from organ ischemia due to static or dynamic malperfusion from the onset, we first perform OAR or TEVAR in accordance with the treatment algorithm. If ischemia persists after interventions, additional laparotomy or stent implantation of the visceral arteries is performed in the acute phase.

11. Lastly, Almost All Patients are Managed with OMT Alone

As part of this strategy, the three treatment modalities, OAR, TEVAR, and OMT, are rationally combined, and only patients who require the necessary treatment receive it in a timely manner, excluding overtreatment or undertreatment. In almost all cases, thoracoabdominal aortic replacement is unnecessary, and almost all patients are ultimately managed with OMT alone (Figure S1). Rigorous annual CT follow-up is also continued. This tailor-made, patient-centered treatment strategy may be suitable for acute aortic dissection.

From both ethical and clinical perspectives, it may be more appropriate to accumulate evidence from consecutive cases treated under a consistent, individualized strategy, one that applies intervention only when clinically justified and refrains from it when not.

Funding: This study received no external funding.

Conflicts of Interest: The authors declare no conflicts of interest.

Abbreviations.

The following abbreviations are used in this manuscript:

CPB	Cardiopulmonary bypass
CT	Computed tomography
CTAG	Conformable thoracic aortic graft
dSINE	Distal stent graft-induced new entry
FET	Frozen elephant trunk
FLE	False lumen expansion
IRAD	International Registry of Aortic Dissection
IVUS	Intravascular ultrasound
OAR	Open aortic repair
OMT	Optimal medical treatment
RTAD	Retrograde type A aortic dissection
SCP	Selective cerebral perfusion
TEVAR	Thoracic endovascular aortic repair

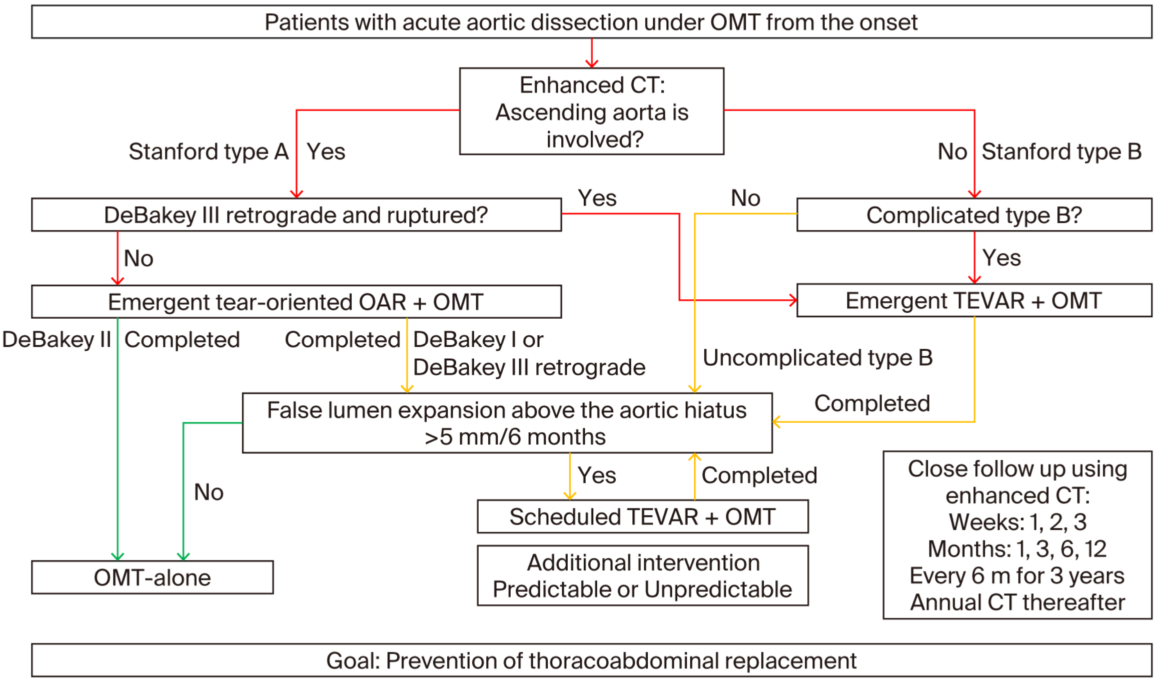

Figure S1. Treatment algorithm of the strategy to maximize effectiveness and minimize risk. Red arrows: Quick decision-making is needed. Yellow arrows: Continue to monitor closely and make a decision if necessary. Green arrows: Stable and close follow-up is needed. CT, computed tomography; OAR, open aortic repair; OMT, optimal medical treatment; TEVAR, thoracic endovascular aortic repair.

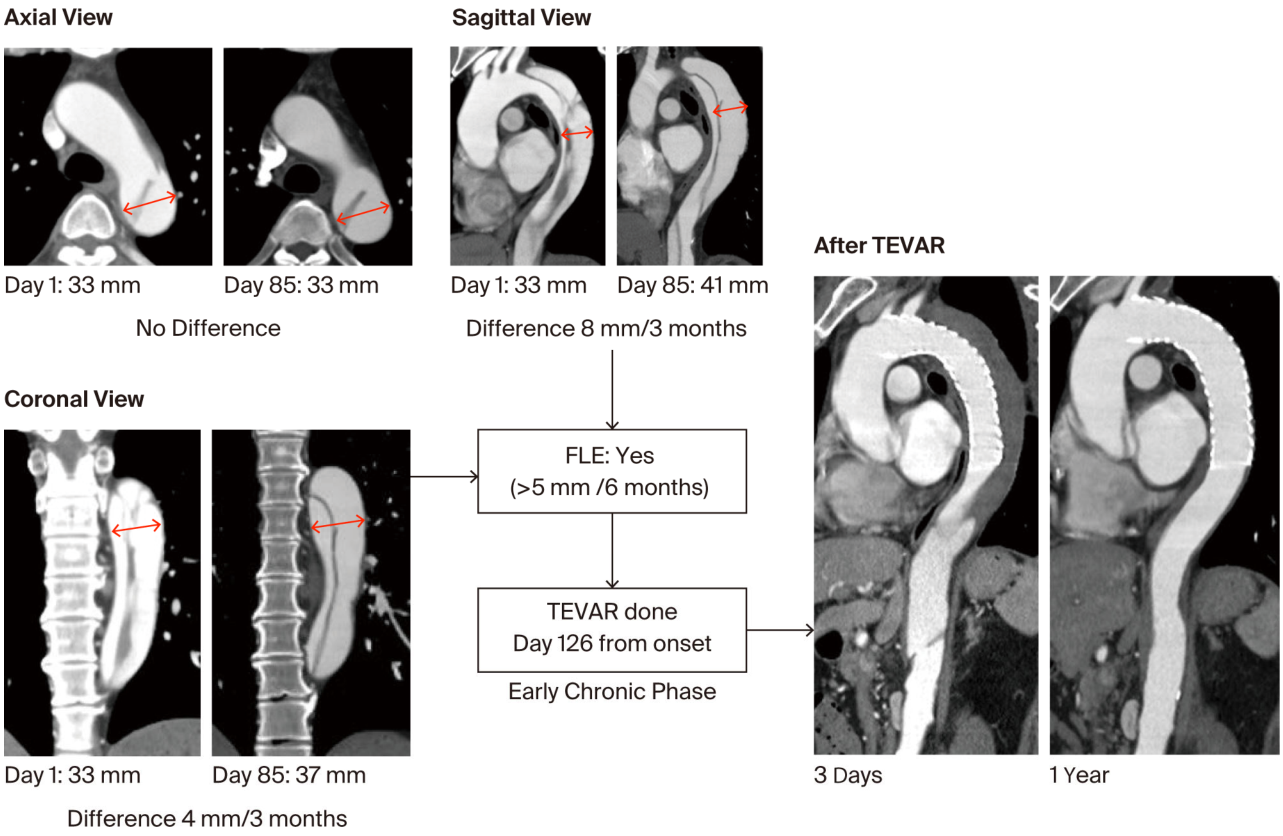

Figure S2. Assessment of false lumen expansion (FLE) and postoperative course after TEVAR. Although no expansion was observed in the axial view, significant expansion was observed in the sagittal and coronal views, and false lumen expansion was confirmed. TEVAR was performed 126 days after onset, and the postoperative course was favorable. TEVAR, thoracic endovascular aortic repair.

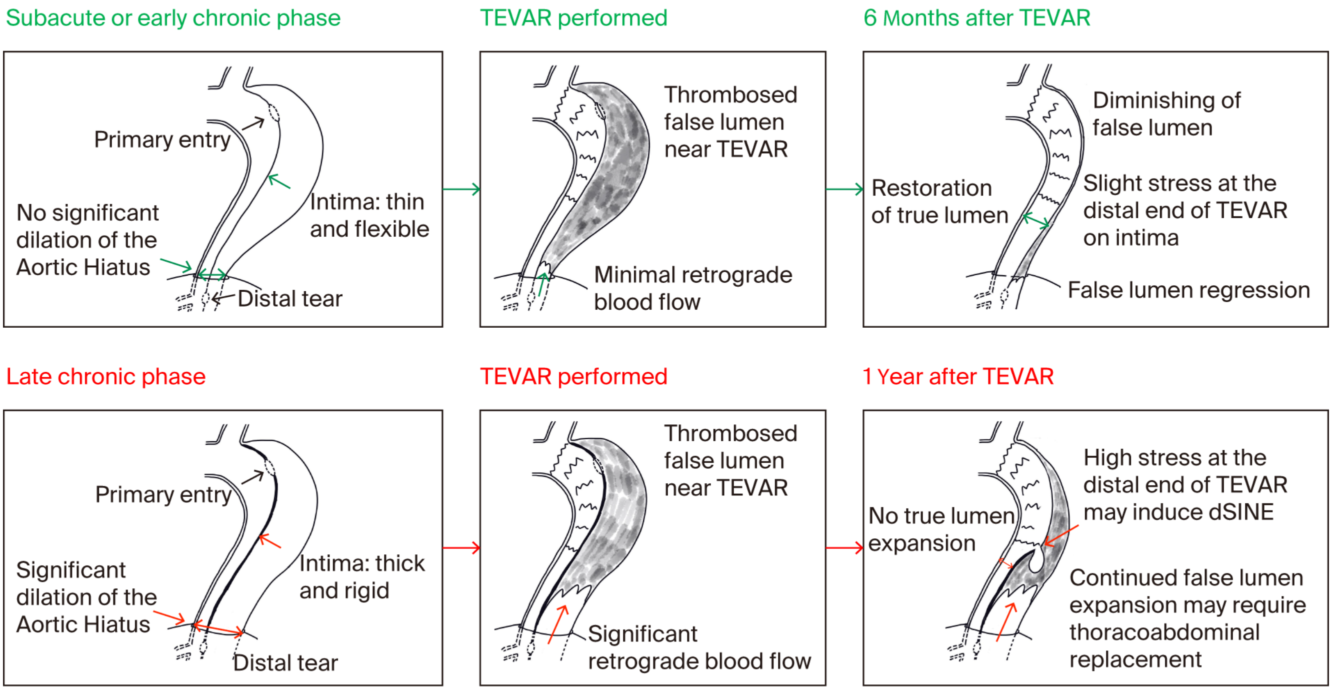

Figure S3. Aortic dissection progression and TEVAR timing based on the aortic hiatus. The upper figure shows TEVAR performed during the subacute or early chronic phase, and the lower figure depicts TEVAR performed in the late chronic phase. It is recommended to conduct TEVAR before significant dilation of the aortic hiatus occurs and the intima loses flexibility. dSINE, distal stent-induced new entry; TEVAR, thoracic endovascular aortic repair.
